# Warfarin and the Risk of Death, Stroke, and Major Bleeding in Patients With Atrial Fibrillation Receiving Hemodialysis: A Systematic Review and Meta-Analysis

**DOI:** 10.3389/fphar.2018.01218

**Published:** 2018-11-06

**Authors:** Hong Lei, Li-Ting Yu, Wei-Ning Wang, Shun-Guo Zhang

**Affiliations:** ^1^Department of Traditional Medicine Testing, Institute for Drug and Instrument Control of Beijing Military Area Command, Beijing, China; ^2^Department of Clinical Pharmacy, Shanghai Children's Medical Center, Shanghai Jiao Tong University School of Medicine, Shanghai, China

**Keywords:** warfarin, atrial fibrillation, hemodialysis, anticoagulation therapy, stroke

## Abstract

**Background:** Up to date, the efficacy and safety of warfarin treatment in atrial fibrillation patients receiving hemodialysis remain controversial. So we performed this meta-analysis to try to offer recommendations regarding warfarin management in this population.

**Methods:** We searched Pubmed, Embase, and Cochrane library and reviewed relevant reference lists from 1980 to March 2018. Studies were included if they described the risks of mortality, stroke, and bleeding events with or without warfarin in atrial fibrillation patients receiving hemodialysis.

**Results:** Overall, the use of warfarin was not associated with mortality (OR = 0.95, 95%CI = 0.89–1.02), stroke (OR = 1.06, 95% CI = 0.87–1.30) and ischemic stroke (OR = 0.85, 95% CI = 0.68–1.05), but its use could increase the risks of hemorrhagic stroke (OR = 1.34, 95% CI = 1.13–1.59) and major bleeding (OR = 1.24, 95% CI = 1.14, 1.35). In subgroup analyses, when analyses were mainly restricted to atrial fibrillation patients who were undergoing hemodialysis and taking other anticoagulation agents, warfarin therapy didn't reduce the risks for mortality (OR = 0.98, 95% CI = 0.68–1.42) and ischemic stroke (OR = 1.03, 95% CI = 0.89–1.19), but significantly increased the risks of stroke (OR:1.14, 95% CI = 1.01–1.29) and bleeding events such as hemorrhagic stroke (OR = 1.42, 95% CI = 1.14–1.77) and major bleeding (OR = 1.24, 95% CI = 1.14–1.35). While in patients who didn't take other anticoagulation agents or aspirin, warfarin use was not associated with all-cause mortality (OR = 0.90, 95% CI = 0.78–1.04), or any stroke (OR = 1.00, 95% CI = 0.71–1.40). Its use was associated with significantly decreased risk of ischemic stroke (OR = 0.71, 95% CI = 0.60–0.85), but not associated with hemorrhagic stroke (OR = 1.45, 95% CI = 0.83–2.55). Besides, another subgroup analysis showed that warfarin therapy didn't exert a protective role in patients with normal serum lipid levels (OR = 1.04, 95% CI = 0.85–1.26), but seemed to decrease the risk of ischemic stroke in patients with hyperlipidemia (OR = 0.38, 95% CI = 0.11–1.29).

**Conclusion:** Our results suggested that it was necessary to prescribe warfarin for the prevention of ischemic events in hemodialysis patients with atrial fibrillation, but if these patients were already prescribed with other anticoagulants for the treatment of other co-existing diseases, then warfarin was not recommended.

## Introduction

Although warfarin was indicated in the general atrial fibrillation (AF) population, its efficacy and safety in hemodialysis patients with atrial fibrillation remained controversial (Wizemann et al., 2010; Hori et al., 2013). It is well-known that hemodialysis patients have higher risk of bleeding since the routine practice of hemodialysis requires systemic anticoagulation with heparin (Mitsuma et al., 2016). When warfarin was prescribed, the bleeding risk could be aggravated by the combination of these two drugs. Thus, how to balance the risk-benefit of warfarin therapy for prevention of embolic events in patients with AF receiving hemodialysis was of clinical significance. The latest clinical guidelines recommended the warfarin therapy in these patients when the patient's score on CHADS_2_ was more than 2 (Chan et al., 2016). However, several observational studies, which mainly enrolled patients with two or more CHADS_2_ scores, produced conflicting results concerning the benefit and safety of warfarin therapy (Olesen et al., 2012; Tan et al., 2017). Hence the role of warfarin in AF patients receiving hemodialysis still needed further investigation.

Recent meta-analyses demonstrated that warfarin therapy could not provide a protective effect for stroke prevention, but may increase the risk of major bleeding in this particular population (Li et al., 2015; Nochaiwong et al., 2016). Although their conclusions were consistent, they still failed to offer meaningful suggestions to clinical practices because of two reasons: Firstly, the lack of evidence from randomized controlled trials in these populations; secondly, the confounding factors responsible for heterogeneity between studies were not identified. Therefore, we conducted an updated meta-analysis to reexamine the efficacy and safety of warfarin in AF patients receiving hemodialysis and assessed potential confounding factors affecting the risk-benefit profile of warfarin in this population.

## Methods

### Research question

This review assessed the risk-benefit profile of warfarin in AF patients receiving hemodialysis.

### Data sources and searches

We searched Medline, the Cochrane Central Register of Controlled Trials, and Embase using following terms: “atrial fibrillation,” “end stage renal disease,” “hemodialysis,” “warfarin,” “anticoagulation.” All databases were searched from their start date to Dec 10, 2017. There were no language limitations for the initial search.

### Study selection

#### Inclusion criteria

Studies examining the benefit and safety of warfarin in hemodialysis patients with AF were included. Studies needed to report the crude event data on the risk for any of the following events: death, stroke, major bleeding. Studies that reported only ratios (i.e., hazard ratios) for our outcomes of interest were excluded, because these data couldn't be extracted for the following analyses.

#### Study selection

Two reviewers (Hong Lei and Li-Ting Yu) independently screened the titles and abstracts of the articles. Included studies were reviewed by the same 2 reviewers for data extraction. In case of disagreement, a third reviewer (Wei-Ning Wang) was consulted for the decision on inclusion or exclusion for full-text evaluation.

### Data extraction and quality assessment

The following data were extracted from the included studies: The types of study population, follow-up time, mean age, the ratio of female, history of some cardiovascular diseases (stroke, hypertension, and hyperlipidemia). Also it was noted whether other anticoagulation drugs were prescribed to these patients. Each included study only provided study level data (baseline patient characteristics) rather than individual patient data, which may limit the analysis in certain groups of patients.

The study quality was judged using Newcastle-Ottawa Scale for cohort studies as previously described (Stang, 2010).

### Data analysis

The significance of the combined odds ratio (OR) was determined by the *Z*-test, in which *P* < 0.05 was considered significant. The χ2-based Q statistical test was used for the assessment of the between-study heterogeneity, which was considered significant for *P* < 0.1. The degree of inconsistency was estimated by *I*^2^ statistics. The *I*^2^ value indicated low (< 25%), moderate (25%−75%) and high (>75%) homogeneity. In analyses, if the heterogeneity was low, then we used a fixed-effect model, or else applied the random-effect model. Software of Review Manager 5.3 was used to perform the meta-analyses (available from Cochrane). To explore the sources of heterogeneity, subgroup analysis was performed. These subgroups were based on patient characteristics.

## Results

**Figure 1:**
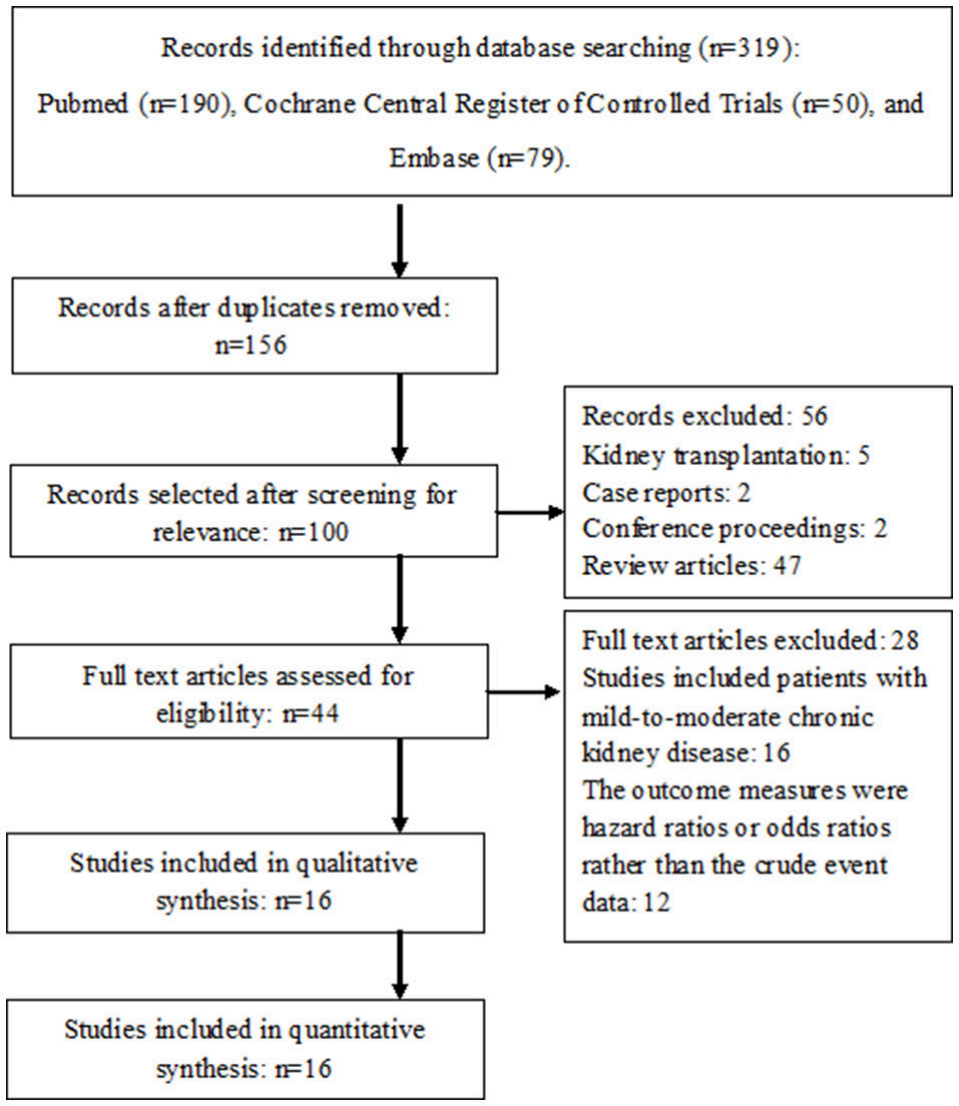
Flow diagram of selected studies.

A total of fifteen studies were finally included in this meta-analysis (Figure 1 Chan et al., 2009, 2015; Lai et al., 2009; Winkelmayer et al., 2011; Wakasugi et al., 2013; Chen et al., 2014; Genovesi et al., 2015; Shen et al., 2015; Yodogawa et al., 2015; Brancaccio et al., 2016; Garg et al., 2016; Mitsuma et al., 2016; Wang et al., 2016; Yamashita et al., 2016; Kai et al., 2017; Yoon et al., 2017). They were all cohort studies. There were no randomized controlled studies that evaluated the influence of warfarin therapy in AF patients receiving hemodialysis.

### Characteristics of the studies

The characteristics of the included articles are reported in Table 1 (see below).

**Table 1:** Characteristics of 16 studies included in the review.

**Author (Year)**	**Study duration (Years)**	**Control group**	**Mean age (years)**	**% With stroke history**	**% Female**	**% with hypertension/hyperlipidemia**	**Stroke risk stratification (score; % with high risk)**	**Bleeding risk stratification (score; % with high risk)**
Kai et al., 2017	9	No anticoagulation	69.2	24.0	38.4	99.2/92.9	CHA2DS2-VASc score (≧2: 98.8%)	HAS-BLED score (≧3: 98.5%)
Yodogawa et al., 2015	4	40% anti-PLT	69.5	10.0	20.0	57.0/NR	CHADS2 score (1.7 ± 1.1)	NR
Chan et al., 2009	1.6	No anticoagulation	72.6	14.4	42.2	79.7/NR	CHADS2 score (2.75 ± 0.05)	NR
Chan et al., 2015	2	100% Aspirin	70.6	12.7	38.8	NR/NR	CHADS2 score (2.4 ± 1.0)	Bleeding index score (1.9 ± 0.6)
Chen et al., 2014	4	No anticoagulation	NR	5.1	58.5	81/32	NR	NR
Lai et al., 2009	2.6	44% ASP	73	8	29	64/67	NR	NR
Wang et al., 2016	10	No anticoagulation	59.8	NR	39.0	98.3/NR	CHA2DS2-VASc score (3.7 ± 1.6)	NR
Brancaccio et al., 2016	1.1	No anticoagulation	71.9	17	39	NR/NR	NR	NR
Winkelmayer et al., 2011	2	No anticoagulation	68.9	NR	58.7	82.7/NR	NR	NR
Shen et al., 2015	1.1	No anticoagulation	61.2	22	50.3	97.2/NR	CHADS2 score (≧2: 90.2%)	HAS-BLED score (≧3: 63.3%)
Genovesi et al., 2015	2	80% anti-PLT	NR	15.7	35.8	76.1/36.6	CHA2DS2-VASc score (≧2: 95.9%)	HAS-BLED score (≧2: 99%)
Wakasugi et al., 2013	4	No anticoagulation	67.8	14.0	43.0	46/NR	CHADS2 score (≧2: 86.7%)	NR
Yoon et al., 2017	1.3	86% anti-PLT	67.8	NR	40.1	89.4/48.1	CHA2DS2-VASc score (≧2: 87%)	HAS-BLED score (≧3: 75.2%)
Yamashita et al., 2016	2.8	No anticoagulation	74.4	20.0	38.0	78.0/35.0	CHADS2 score (2.5 ± 1.4) CHA2DS2-VASc score (3.4 ± 1.7)	NR
Garg et al., 2016	5.0	68.3% ASP	75.0	20.1	44.6	84.8/NR	CHA2DS2-VASc score (≧2: 100%)	HAS-BLED score (≧2: 98.3%)
Mitsuma et al., 2016	2.0	36% ASP	71.2	27.0	29	53.0/14.0	CHADS2 score (≧2: 55%)	HAS-BLED score (≧3: 87%)

*ASP, aspirin; anti-PLT, anti-Platelet drugs*.

### Assessment of methodological quality

The NOS quality scores of all the included studies were high, ranging from 7 to 9 point (Table 2).

**Table 2:** Assessment of methodological quality by NOS.

**Study**	**Selection**				**Comparability**	**Outcome**			**Total**
	**Exposed cohort representativeness**	**Non exposed cohort selection**	**Ascertainment of exposure**	**Outcome not present at start of study**	**Comparability of cohorts**	**Assessment of outcome**	**Follow-up long enough**	**Adequacy of follow up**
Kai et al., 2017	▴	▴	▴	▴	▴▴	▴	▴	▴	9
Yodogawa et al., 2015	▴	▴	▴	▴	▴	▴	▴	▴	8
Chan et al., 2009	▴	▴	▴	▴	▴▴	▴	▴	▴	9
Chan et al., 2016	▴	▴	▴	▴	▴▴	▴		▴	8
Chen et al., 2014	▴	▴	▴	▴	▴▴	▴	▴	▴	9
Lai et al., 2009	▴		▴	▴	▴	▴	▴	▴	7
Wang et al., 2016	▴	▴	▴	▴	▴	▴	▴	▴	8
Brancaccio et al., 2016	▴	▴	▴	▴	▴▴	▴		▴	8
Winkelmayer et al., 2011	▴	▴	▴	▴	▴▴	▴	▴	▴	9
Shen et al., 2015	▴	▴	▴	▴	▴▴	▴		▴	8
Genovesi et al., 2015	▴	▴	▴	▴	▴	▴	▴	▴	8
Wakasugi et al., 2013	▴	▴	▴	▴	▴▴	▴	▴	▴	9
Yoon et al., 2017	▴	▴	▴	▴	▴	▴		▴	8
Yamashita et al., 2016	▴	▴	▴	▴	▴	▴	▴	▴	8
Garg et al., 2016	▴	▴	▴	▴	▴	▴	▴	▴	8
Mitsuma et al., 2016	▴	▴	▴	▴	▴	▴	▴	▴	8

### Mortality, stroke and major bleeding outcomes

**Figure 2:**
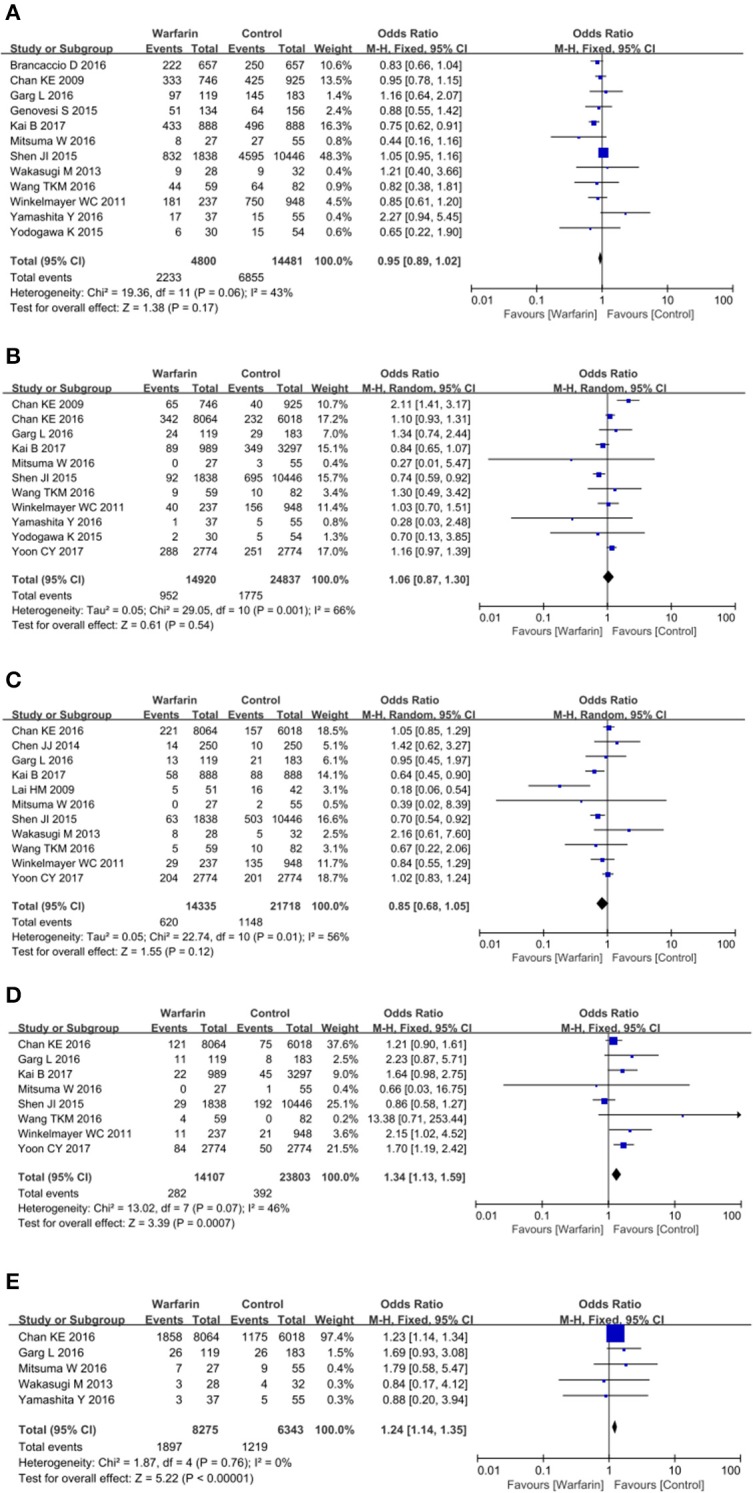
Warfarin use and the risk of mortality **(A)**, all-type stroke **(B)**, ischemic stroke **(C)**, hemorrhagic stroke **(D)**, and major bleeding **(E)** in atrial fibrillation patients receiving hemodialysis.

For mortality, the pooled OR showed no significant association between the warfarin users and non-warfarin users: OR = 0.95 (95% CI = 0.89–1.02; *p* = 0.06; Figure 2A; Table 3). Similarly, no association of stroke among warfarin users and non-warfarin users was found. The pooled OR was 1.06 (95% CI = 0.87–1.30; Figure 2B; Table 3). Although warfarin had no statistically significant effects on all type strokes, its effects on ischemic stroke and hemorrhagic stroke still needed to be identified, respectively since the reduction in occlusive events might be offset by any increase in cerebral bleeds. Surprisingly, warfarin therapy was not associated with a deceased risk of ischemic stroke (OR = 0.85, 95% CI = 0.68–1.05; *p* = 0.01; Figure 2C; Table 3), but associated with an increased risk of hemorrhagic stroke (OR = 1.34, 95% CI = 1.13–1.59; *p* = 0.07; Figure 2D; Table 3), which demonstrated that AF patients receiving hemodialysis could not benefit from warfarin therapy, on the contrary, they would be exposed to a higher risk of cerebral bleeding. Besides, the meta analysis showed that warfarin increased the risk of major bleeding as well (OR = 1.24, 95% CI = 1.14–1.35; *p* = 0.76; Figure 2E; Table 3).

**Table 3:** Odds ratio of clinical outcomes comparing warfarin users vs. non-warfarin users in different populations.

**Outcomes**	**Control group**		**Total**
	**Anti-PLT^1^ or Anti-ASP^2^**	**No anticoagulation**
Death	0.98 (0.68, 1.42)	0.90 (0.78, 1.04)	0.95 (0.89, 1.02)
Stroke	1.14 (1.01, 1.29)	1.00 (0.71,1.40)	1.06 (0.87, 1.30)
IS	1.03 (0.89, 1.19)	0.71 (0.60, 0.85)	0.85 (0.68, 1.05)
HS	1.42 (1.14, 1.77)	1.45 (0.83, 2.55)	1.34 (1.13, 1.59)
Major Bleeds	1.24 (1.14,1.35)	1.22 (0.56, 2.65)	1.24 (1.14, 1.35)

*ASP, aspirin; anti-PLT, anti-Platelet drugs*.

Taken together, observational studies included in this meta-analysis suggested that warfarin was not associated with mortality, stroke and ischemic stroke, but significantly increased the risk of hemorrhagic stroke and major bleeding.

### Subgroup analysis

**Figure 3:**
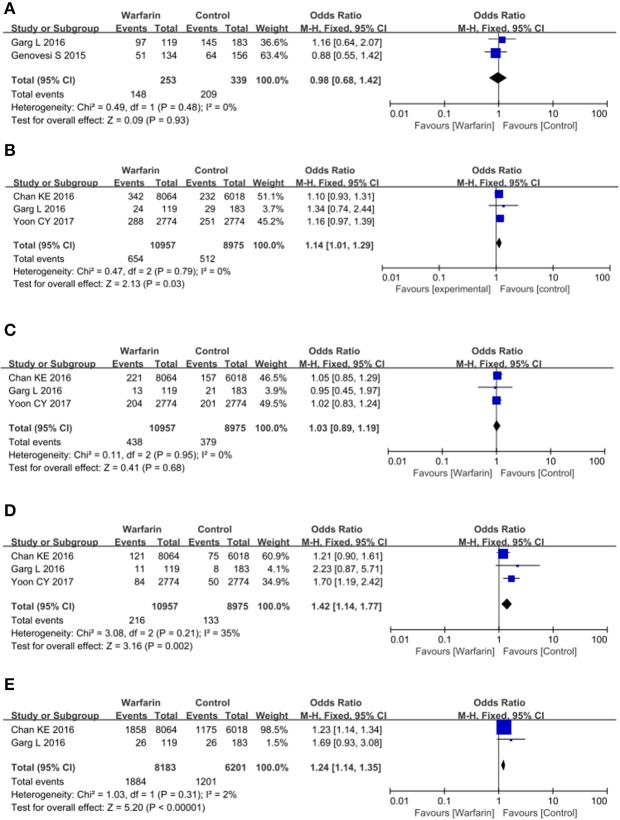
Warfarin use and the risk of mortality **(A)**, all-type stroke **(B)**, ischemic stroke **(C)**, hemorrhagic stroke **(D)**, and major bleeding **(E)** in atrial fibrillation patients receiving hemodialysis who took other anticoagulation drugs in the meantime.

Since there were high statistical heterogeneities among included studies that examined the mortality, stroke, and ischemic stroke, we planned subgroup analysis to assess the potential sources of heterogeneity based on baseline patient characteristics. Subgroup analyses stratified according to the age and history of stroke were performed and they failed to show associations between the use of warfarin and the risks of the outcomes above. We then assessed the source of heterogeneity by the baseline use of anticoagulants and antiplatelet agents and examined two subgroups according to the percents of patients taking anticoagulation agents in our analysis: subgroup 1 (>65%) and subgroup 2 (< 65%). Among these studies, a total of four studies were included in the subgroup 1 (Chan et al., 2015; Genovesi et al., 2015; Garg et al., 2016; Yoon et al., 2017), while the others were included in the subgroup 2 (Chan et al., 2009; Lai et al., 2009; Winkelmayer et al., 2011; Wakasugi et al., 2013; Chen et al., 2014; Shen et al., 2015; Yodogawa et al., 2015; Brancaccio et al., 2016; Mitsuma et al., 2016; Wang et al., 2016; Yamashita et al., 2016; Kai et al., 2017). Of note, when analyses were mainly restricted to AF patients who were undergoing hemodialysis and taking other anticoagulation agents (subgroup 1), the heterogeneity of these analyses were much reduced and the results suggested that warfarin was more likely to do harm than good. There was no association in all-cause mortality among warfarin users and non-warfarin users. The pooled OR was 0.98 (95% CI = 0.68–1.42; *p* = 0.48; Figure 3A; Table 3). For stroke, pooled OR between warfarin users and non-warfarin users was 1.14 (95% CI = 1.01–1.29; *p* = 0.79; Figure 3B; Table 3), which suggested a positive association between the warfarin therapy and the risk of stroke. Since there were two types of stroke and the effects of warfarin on both strokes remained unknown, we examined the impacts of warfarin use on the risk of ischemic stroke and hemorrhagic stroke, respectively. Surprisingly, warfarin therapy was not associated with a decreased risk of ischemic stroke (OR = 1.03, 95% CI = 0.89–1.19; *p* = 0.95; Figure 3C), but associated with a significant increased risk of hemorrhagic stroke (OR = 1.42, 95% CI = 1.14–1.77; *p* = 0.21; Figure 3D; Table 3). Besides, warfarin therapy was associated with a higher risk of major bleeding as well (OR = 1.24, 95% CI = 1.14–1.35; *p* = 0.31; Figure 3E; Table 3).

**Figure 4:**
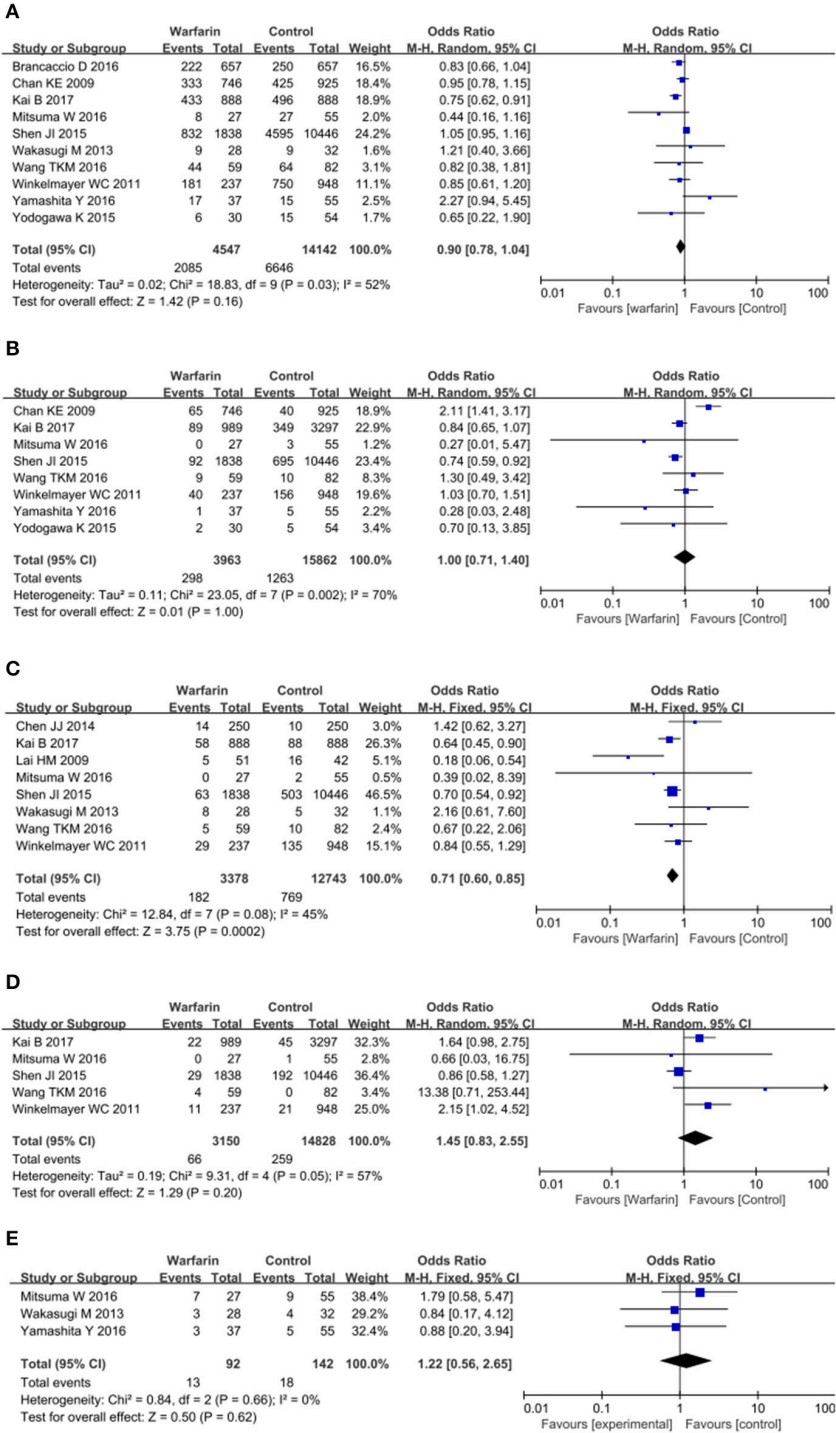
Warfarin use and the risk of mortality **(A)**, all-type stroke **(B)**, ischemic stroke **(C)**, hemorrhagic stroke **(D)**, and major bleeding **(E)** in atrial fibrillation patients receiving hemodialysis who didn't took other anticoagulation drugs.

When most of these patients didn't take other anticoagulation agents or aspirin, they seemed to benefit from warfarin therapy. Although there was no association in all-cause mortality (OR = 0.90, 95% CI = 0.78–1.04; *p* = 0.03; Figure 4A; Table 3) and stroke among warfarin users and non-warfarin users (OR = 1.00, 95% CI = 0.71–1.40; *p* = 0.002; Figure 4B; Table 3), warfarin therapy did decrease the risk of ischemic stroke in these patients and had no negative impact on hemorrhagic stroke. The pooled OR for ischemic stroke was 0.71 (95% CI = 0.60–0.85; *p* = 0.08; Figure 4C; Table 3) and for hemorrhagic stroke was 1.45 (95% CI = 0.83–2.55; *p* = 0.05; Figure 4D). Besides, major bleeding was not significantly increased in patients who received warfarin treatment as well (OR = 1.22, 95% CI = 0.56–2.65; *p* = 0.66; Figure 4E; Table 3).

**Figure 5:**
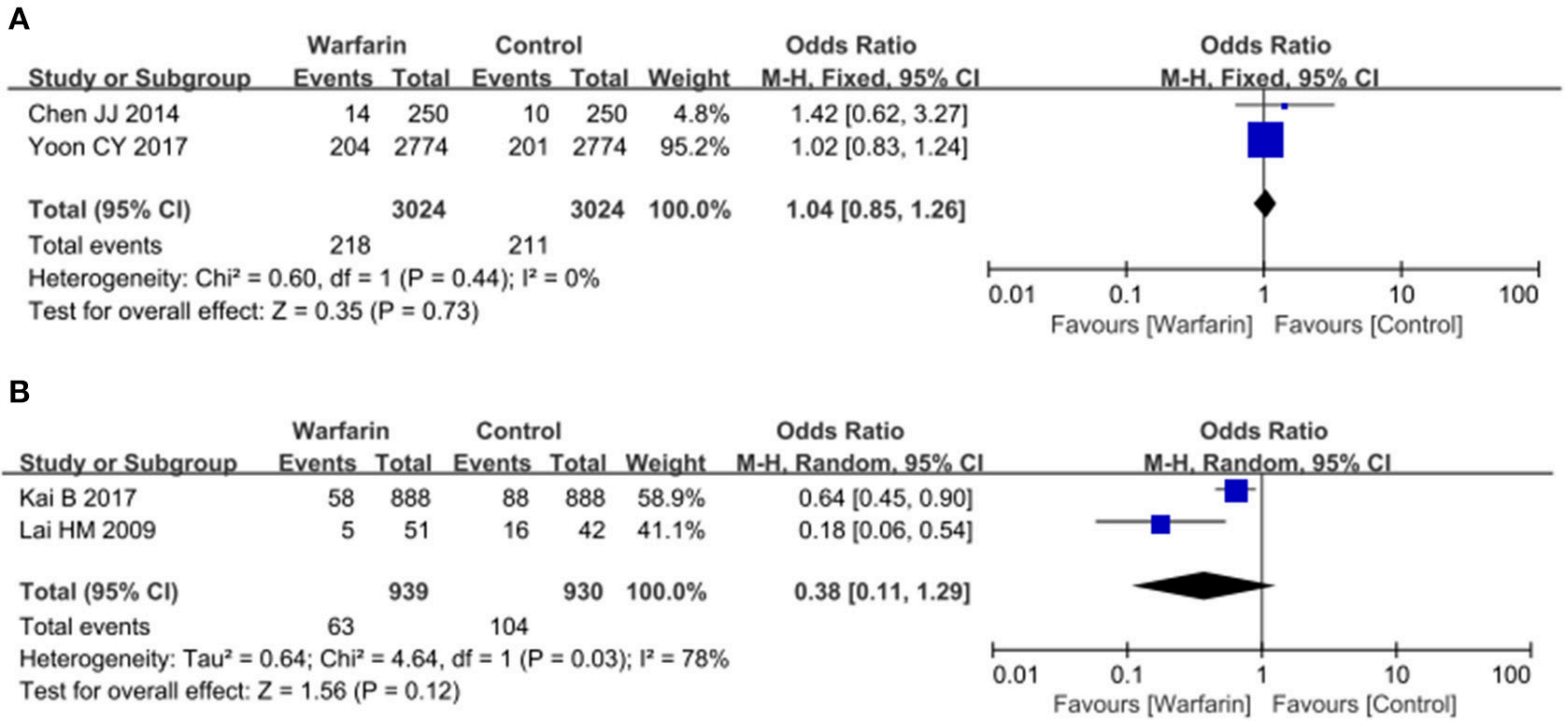
Subgroup analysis of warfarin use and the risk of ischemic stroke in atrial fibrillation patients receiving hemodialysis. Results were presented for patients with almost normal lipid level **(A)** and with hyperlipidemia **(B)**.

Additionally, we tested the effects of warfarin therapy on patients with different serum lipid levels. Since patient level data could not be obtained and we couldn't perform a subgroup analysis according to their lipid concentrations. Thus, we examined two subgroups according to the percents of patients with hyperlipidemia in our analysis: subgroup 1 (< 50%) and subgroup 2 (>65%). However, only six studies provided data on the percents of patients with hyperlipidemia and several preplanned subgroups analyses could not be performed because of limited number of included studies (Lai et al., 2009; Chen et al., 2014; Genovesi et al., 2015; Yamashita et al., 2016; Kai et al., 2017; Yoon et al., 2017). Only one subgroup analysis was performed to test the effects of warfarin therapy on ischemic stroke in these patients. Our results demonstrated that warfarin didn't exert a protective role in the patients with almost normal lipid levels (OR = 1.04, 95% CI = 0.85–1.26; *p* = 0.44; Figure 5A), but seemed to decrease the risk of ischemic stroke in patients with hyperlipidemia although the difference was not significant (OR = 0.38, 95% CI = 0.11–1.29; *p* = 0.03; Figure 5B).

### Risk of bias

The risk of bias was assessed using Newcastle-Ottawa Scale for cohort studies. In total, all the included studies were considered to have a low risk of bias.

## Discussion

### Summary of main findings

Our meta-analysis showed that hemodialysis patients with atrial fibrillation could not benefit from warfarin therapy, since it didn't decrease the risk of ischemic stroke, on the contrary, it did increase the risk of bleeding events such as hemorrhagic stroke and major bleeding. These findings were consistent with previous results of meta-analyses demonstrated by Nochaiwong et al. (2016) and Tan et al. (2016). However, their results were inconclusive due to high heterogeneity among included studies, which made their conclusion less convincing. Although they performed subgroup analysis to explore potential sources of heterogeneity, the definite source of heterogeneity was sample size. They found the use of warfarin was associated with decreased risk of ischemic stroke in smaller studies (size < 500 patients), not in larger studies (size >500 patients). Whether the patient characteristics modified the impact of warfarin use was unknown. The subgroup analyses stratified according to baseline characteristics were not performed either due to limited statistical power or to lack of patient-level data.

Although there were high heterogeneities in our included studies as well, we explored the sources of heterogeneity and finally identified concomitant use of other anticoagulation agents as a potential source of such heterogeneity. If these patients didn't take other anticoagulation drugs, they could benefit from warfarin therapy since it significantly decreased the risk of ischemic stroke without increasing the risk of bleeding events. However, when these patients took aspirin or other anti-Platelet medications due to the con-morbidities, concomitant use of warfarin couldn't decrease the risk of ischemic stroke, on the contrary, it did significantly increase the risk of stroke and severe bleeding events such as hemorrhagic stroke and major bleeding. These findings suggested that it was necessary to prescribe warfarin for the prevention of ischemic events in hemodialysis patients with atrial fibrillation, but if these patients were already prescribed with other anticoagulants for the treatment of other co-existing diseases, then warfarin was not recommended. In other words, taking two or three anticoagulants were not recommended in these patients.

Besides, hyperlipidemia might be another source of heterogeneity of clinical outcomes in these patients. However, this conclusion was less convincing due to limited studies. Totally, Only 6 studies provided data on the percentage of hyperlipidemia patients in the baseline patient characteristics (Lai et al., 2009; Chen et al., 2014; Genovesi et al., 2015; Yamashita et al., 2016; Kai et al., 2017; Yoon et al., 2017). Among them, only 4 studies were included in the subgroup analysis to test whether the benefit of warfarin therapy for ischemic stroke prevention was related to abnormal lipid levels (Lai et al., 2009; Chen et al., 2014; Kai et al., 2017; Yoon et al., 2017). Our results demonstrated that warfarin didn't exert a protective role in the patients with almost normal lipid levels, but seemed to decrease the risk of ischemic stroke in patients with hyperlipidemia. Although there was no evidence that elevated blood lipid level was related to the increased risk of ischemic stroke in atrial fibrillation patients receiving hemodialysis, the association between hyperlipidemia and the risk of ischemic stroke in other diseases was clearly identified. For instance, it was reported that hyperlipidemia was a predictive factor for ischemic stroke and stroke severity in systemic lupus erythematosus (Mikdashi et al., 2007). Another study compared the frequency of hyperlipidemia, hypertension, and diabetes in patients with ischemic stroke and found that patients with abnormal lipid levels were more likely to develop ischemic stroke (Abid et al., 2012). A meta-analysis involving 42 trials showed that statin therapy, which mainly decreased the level of cholesterol, significantly decreased the risk of ischemic stroke by 16% (O'regan et al., 2008). These situations might elucidate the increased benefit of warfarin for ischemic stroke prevention in these hyperlipidemia patients.

Generally, CHADS_2_/CHA_2_DS_2_VAS_C_ scoring system was frequently used to guide toward oral anticoagulation therapy. As recommended by several guidelines, patients who had CHADS_2_/CHA_2_DS_2_VAS_C_≧2 should initiate warfarin treatment (Kim et al., 2016; Chapman et al., 2017). However, among 12 included studies in our meta-analysis, which mainly enrolled patients who had CHADS_2_/CHA_2_DS_2_VAS_C_≧2, there was not a consistent conclusion about the risk-benefit profile of warfarin in these patients (Chan et al., 2009, 2015; Winkelmayer et al., 2011; Wakasugi et al., 2013; Genovesi et al., 2015; Shen et al., 2015; Yodogawa et al., 2015; Garg et al., 2016; Mitsuma et al., 2016; Yamashita et al., 2016; Kai et al., 2017; Yoon et al., 2017). Only 2 studies showed that the receipt of warfarin was associated with a significantly lower risk of ischemic stroke, while others failed to prove the association between the warfarin therapy and clinical outcomes (Shen et al., 2015; Kai et al., 2017). Such differences across studies were probably related to the low cutoff points of CHADS_2_/CHA_2_DS_2_VAS_C_ scoring system since a recent report from China showed that warfarin therapy may be suitable for atrial fibrillation patients receiving hemodialysis who had CHADS_2_≧4 or CHA_2_DS_2_VAS_C_ ≧6 (Chao et al., 2014). Another potential explanation for this inconsistency was that there might be other potential confounders which can bias these results as well. If we mainly enrolled patients who didn't take other anticoagulation drugs other than warfarin, then these patients with a CHADS_2_/CHA_2_DS_2_VAS_C_ score of more than 2 could benefit from warfarin therapy since it could decrease the risk of ischemic stroke by nearly 30% without increasing the risk of major bleeding events (data not shown). This finding suggested that concomitant use of other anticoagulation drugs could be a confounding factor, which could affect the predictive value of CHADS_2_/CHA_2_DS_2_VAS_C_ scoring system in identifying groups of high risk hemodialysis patients with atrial fibrillation.

Furthermore, advanced age was considered as another dependent risk factor that was linked to elevated risk of adverse clinical events induced by warfarin therapy. Wizemann et al. found that for atrial fibrillation patients receiving hemodialysis who were more than 75 years old, the risk of anticoagulation therapy might outweigh the benefit (Wizemann et al., 2010). However, we didn't find the modification of the effect of warfarin by age in our meta-analysis. The average age of patients in our included studies ranged from 59.8 to 75 years, and there was not a trend indicating higher risk of adverse clinical events among elderly patients treated with warfarin (data not shown). Therefore, advanced age might have limited roles in guiding anticoagulation therapy in these patients.

### Strengths and limitations

This study has several strengths. Firstly, previous meta-analysis demonstrated that hemodialysis patients with atrial fibrillation could not benefit from warfarin therapy, however their conclusions were less convincing due to high heterogeneities. Although there were high heterogeneities in our included studies as well, we explored the sources of heterogeneity and finally identified concomitant use of other anticoagulation agents as a potential source of such heterogeneity. This finding emphasized the importance of warfarin prescription in the treatment of atrial fibrillation patients receiving hemodialysis despite of complex interrelationship between the pathophysiology and the clinical outcomes in these patients. Secondly, we demonstrated for the first time that these patients with hyperlipidemia were more likely to receive benefit from warfarin than those with normal lipid levels, though more studies were needed to confirm it.

We acknowledge several limitations of our study as well. Firstly, since there are no randomized controlled trials comparing anticoagulation treatments in atrial fibrillation patients receiving hemodialysis up to now, our findings should still be interpreted with great caution. Secondly, using study level data rather than individual patient data limited analysis in certain groups of patients. Access to individual patient data about the co-medication history helped to better clarify whether concomitant use of other anticoagulation drugs would cause more harm than good in the subgroup of patients. Thirdly, a total of four included studies didn't mention the CHADS_2_ or CHA_2_DS_2_-VASc score in the baseline characteristics and one study enrolled patients with an average CHADS_2_ score of 1.7 (< 2). These low risk or unknown risk patients might be poor candidates for warfarin therapy.

## Conclusion

In conclusion, our study has demonstrated that atrial fibrillation patients receiving hemodialysis could benefit from warfarin treatment, but if other anticoagulation drugs were prescribed already due to co-existing diseases, then warfarin was not recommended. Traditional risk factors such as high CHADS_2_/CHA_2_DS_2_VAS_C_ score (≧2), could still identify groups of high risk hemodialysis patients with atrial fibrillation if they didn't take other anticoagulation drugs.

## Author contributions

HL and L-TY analyzed the data and wrote the manuscript. W-NW collected the data and performed the analyses. S-GZ designed the study and amended the paper.

### Conflict of interest statement

The authors declare that the research was conducted in the absence of any commercial or financial relationships that could be construed as a potential conflict of interest.
